# Sexual Function in 16- to 21-Year-Olds in Britain

**DOI:** 10.1016/j.jadohealth.2016.05.017

**Published:** 2016-10

**Authors:** Kirstin R. Mitchell, Rebecca Geary, Cynthia Graham, Soazig Clifton, Catherine H. Mercer, Ruth Lewis, Wendy Macdowall, Jessica Datta, Anne M. Johnson, Kaye Wellings

**Affiliations:** a Centre for Sexual Health, London School of Hygiene and Tropical Medicine, London, United Kingdom; b MRC/CSO Social and Public Health Sciences Unit, Institute of Health and Wellbeing, University of Glasgow, Glasgow, United Kingdom; c Department of Infection and Population Health, University College London, London, United Kingdom; d Department of Psychology, University of Southampton, Southampton, United Kingdom; e Department of Sociology, University of the Pacific, Stockton, California

**Keywords:** Young people, Early adulthood, Sexual function problems, Sexual dysfunction, Sexual well-being, Help seeking, Avoidance of sex, Prevalence, Population survey

## Abstract

**Purpose:**

Concern about young people's sexuality is focused on the need to prevent harmful outcomes such as sexually transmitted infections and unplanned pregnancy. Although the benefit of a broader perspective is recognized, data on other aspects of sexuality, particularly sexual function, are scant. We sought to address this gap by measuring the population prevalence of sexual function problems, help seeking, and avoidance of sex in young people.

**Methods:**

A cross-sectional stratified probability sample survey (Natsal-3) of 15,162 women and men in Britain (response rate: 57.7%), using computer-assisted self-interviews. Data come from 1875 (71.9%) sexually active, and 517 sexually inactive (18.7%), participants aged 16–21 years. Measures were single items from a validated measure of sexual function (the Natsal-SF).

**Results:**

Among sexually active 16- to 21-year-old participants, 9.1% of men and 13.4% of women reported a distressing sexual problem lasting 3 months or more in the last year. Most common among men was reaching a climax too quickly (4.5%), and among women was difficulty in reaching climax (6.3%). Just over a third (35.5%) of men and 42.3% of women reporting a problem had sought help, but rarely from professional sources. Among those who had not had sex in the last year, just >10% of young men and women said they had avoided sex because of sexual difficulties.

**Conclusions:**

Distressing sexual function problems are reported by a sizeable minority of sexually active young people. Education is required, and counseling should be available, to prevent lack of knowledge, anxiety, and shame progressing into lifelong sexual difficulties.

**Implications and Contribution**

This nationally representative data from Britain shows that distressing sexual function problems are not uncommon in young people (aged 16–21 years). In sex education and sexual health services, professionals need to acknowledge the importance of sexual well-being and provide opportunities for young people to raise and discuss their concerns.

Professional interest in young people's sexual behavior is most often driven by concern to prevent the harms of sex, primarily unplanned pregnancy and sexually transmitted infection (STI) transmission [1], [2], [3] and, increasingly, nonconsensual sex. Qualitative work suggests that young people themselves are equally concerned with issues affecting their sexual well-being. They may be anxious about their sexual orientation or identity [4], feel social pressure to consent to activities they dislike or find painful [5], or struggle against norms that make it difficult to admit to experiences that are less than ideal [6], [7].

While issues around volition, sexual identity, and sexual reputation have been quite well documented, less is known about problems young people might have with sexual response and function. This is partly because sexual function problems are assumed to be more relevant to older adults. Sexual function is defined as an individual's ability to respond sexually or to experience sexual pleasure [8] and sexual function problems are those that interfere with these. Population prevalence studies of sexual function problems typically include participants as young as 16 or 18 years, but often use broad age categories, up to 29 years [9] and rarely provide specific detail on young people under 24 years [10], [11], [12]. Few studies have focused specifically on early adulthood, and these have not generally used nationally representative data [13], [14].

There is increasing recognition that sexual health should be considered broadly [15], [16], and the holistic definition endorsed by WHO—“a state of physical, emotional, mental and social well-being in relation to sexuality” [17]—is steadily gaining currency. In young people, sexual health includes “positive developmental contributions of sexuality, as well as the acquisition of skills pertinent to avoiding adverse sexual outcomes” [18]. There is evidence that goals relating to sexual satisfaction and pleasure shape both risk taking and risk-reduction practices [16], [19]. For instance, fears about erectile functioning among young men have been shown to contribute to resistance to condom use [20] and to inconsistent use [21]. Good sexual health in adolescents is associated with risk reduction behaviors, such as condom use and sexual abstinence [18], and sexual function in adults is inversely associated with risk behavior [22]. Interventions that safeguard pleasure may therefore be more effective than those that ignore this aspect [16], [23]. The current lack of data on sexual function in young people limits efforts to address sexual health holistically and reinforces the belief that sexual function and well-being are less relevant to prevention interventions targeting young people [1], [24].

We have previously reported on the prevalence of sexual function problems in adults aged 16–74 years using data from the third National Survey of Sexual Attitudes and Lifestyles (Natsal-3) [22]. Here, we use this same data set to address the gap in empirical data on sexual function problems (including those that cause distress), help seeking about one's sex life, and avoidance of sex because of problems, in young people aged 16–21 years in Britain.

## Methods

### Participants and procedure

We present data from 16- to 21-year-old participants in Natsal-3, a stratified probability sample survey of 15,162 men and women aged 16–74 years in Britain, interviewed between September 2010 and August 2012. We focus on the early adulthood period and the early stages of sexual careers before young people “settle” into longer term partnerships and sexual habits. We used a multistage, clustered, and stratified probability sample design, with the U.K. Postcode Address File as the sampling frame and postcode sectors (n = 1,727) selected as a primary sampling unit. Within each primary sampling unit, 30 or 36 addresses were selected at random, and within each household, an eligible adult was selected using a Kish grid. After weighting to adjust for unequal probabilities of selection, the Natsal-3 sample was broadly representative of the British population as described by 2011 Census figures [25].

Participants were interviewed at home by a trained interviewer, using a combination of computer-assisted face-to-face and computer-assisted self-interview (CASI) for the more sensitive questions. The interviewer was present and available to help while participants completed the CASI but did not view answers. At the end of the CASI sections, answers were “locked” into the computer and were inaccessible to the interviewer. The interview lasted for about an hour, and participants received £15 as a token of appreciation. The survey instrument underwent thorough cognitive testing and piloting [26].

The overall response rate was 57.7% of all eligible addresses (64.8% among participants aged 16–44 years). The cooperation rate (proportion of respondents at eligible addresses where contact was made agreeing to take part in the survey) was 65.8%. Details of the survey methodology are published elsewhere [25], [27]. Natsal-3 was approved by the Oxfordshire Research Ethics Committee A. Participants provided oral consent for interviews.

### Outcome measures

Participants reporting vaginal, oral, or anal sex with one or more partner in the past year were classified as “sexually active” and asked whether they had experienced any of a list of eight difficulties with their sex life lasting 3 months or longer in the past year. These were lacked interest in having sex, lacked enjoyment in sex, felt anxious during sex, felt physical pain as a result of sex, felt no excitement or arousal during sex, did not reach a climax (experience an orgasm) or took a long time to reach a climax despite feeling excited or aroused, reached climax (experienced an orgasm) more quickly than you would like, had an uncomfortably dry vagina (asked of women only), and had trouble getting or keeping an erection (asked of men only). For each item, they endorsed (responded yes), participants were then asked how they felt about the problem (response options: not at all distressed; a little distressed; fairly distressed; very distressed). We also asked how long they had experienced the difficulty and how often symptoms occurred (data not presented in this article).

All sexually experienced participants (those who had ever had a sexual experience), regardless of their sexual activity in the last year, were asked to appraise their sex life overall, including whether they had avoided sex because of sexual difficulties experienced by themselves or their partner (agree strongly, agree, neither agree nor disagree, disagree, disagree strongly). Participants agreeing strongly or agreeing were then presented with the same list of problems and asked to indicate which, if any, had caused them to avoid sex. Additional response options were as follows: “my partner had one (or more) sexual difficulty” and “none of these things caused me to avoid sex.” Multiple responses were allowed. Participants were also asked if they felt distressed or worried about their sex life using a five-point Likert scale. Finally, participants were asked whether they had sought help or advice regarding their sex life from any of a list of sources in the last year, and if yes, to select all that apply. These options were subsequently grouped as family member/friend, media/self-help (includes information and support sites on the internet; self-help books/information leaflets; self-help groups; helpline), and professional (includes general practitioner/family doctor; sexual health/genito-urinary medicine/STI clinic; psychiatrist or psychologist; relationship counselor; other type of clinic or doctor), or have not sought any help. These items come from the Natsal-SF; a measure of sexual function specifically designed and validated for use in this and other population prevalence surveys. The 17-item Natsal-SF measure has good fit (comparative fit index = .963; Tucker Lewis index = .951; root mean square error of approximation = .064), can discriminate between clinical and general population groups, and has good test–retest reliability (*r* = .72) [22], [28].

### Statistical analysis

All analyses were done using the complex survey functions of Stata (version 12; StataCorp LP, College Station, TX) to account for the weighting, clustering, and stratification of the data. Analysis was restricted to all sexually experienced men and women aged 16–21 years. Item nonresponse in Natsal-3 was low (almost always <5%, and often 1%–3%), so patients with missing data were excluded from analysis. Among sexually active participants (those reporting at least one sexual partner in the year before interview), we present descriptive statistics for reporting of sexual function problems (lasting 3 or more months in the last year), and the proportion distressed by their problem. We also report the proportion seeking help from the range of sources, stratified by reporting one or more sexual function problem. For participants who were not sexually active in the last year, we report descriptive statistics for three outcomes: sexual satisfaction, distress about sex life, and avoidance of sex because of a sexual difficulty.

## Results

Most men and women (72%) aged 16–21 years reported having one or more sexual partner in the last year and so were categorized as sexually active (854 men and 1,021 women). Table 1 shows the proportion of these men reporting each of eight sexual function problems lasting 3 months or more in the last year. A third of these men (33.8%) experienced one or more sexual function problem (first column of Table 1), and 9.1% reported one or more distressing sexual function problem(s) (second column); implying that among men reporting one or more problem, just over a quarter (26.9%) felt distressed (third column).

Among men, reaching a climax too quickly was the most common problem (13.2%). Just over a third of men with this problem (34.2%) felt distressed about it, making it the most common distressing problem among sexually active 16- to 21-year-old men (4.5%). Difficulty getting and keeping an erection was less commonly reported (7.8%), but more frequently caused distress (among 42.1%) and was thus the second most common distressing problem (by 3.3% of men in the age group). Although lack of interest in sex was the second most commonly reported problem (experienced by 10.5%), only 13.2% of men reporting this problem were distressed by it, and overall, 1.4% experienced it as a distressing problem. Three distressing problems were reported by <1% of sexually active young men: pain, lacking excitement/arousal, and lacking enjoyment.

Table 2 shows the proportion of young sexually active women reporting each sexual function problem, and of those experiencing the problem, the proportion distressed about it. Just under half (44.4%) of these women experienced one or more sexual function problem lasting 3 months or more in the last year, and 13.4% reported a distressing problem; implying that of those reporting one or more problem, just less than a third (30.2%) were distressed.

The most common problems among women were lacking interest in sex (22.0%) and experiencing difficulty in reaching climax (21.3%), and these were also the most common distressing problems (5.3% and 6.3%, respectively). The problems most commonly associated with distress were feeling anxious during sex (34.7%), feeling physical pain as a result of sex (35.9%), and lacking excitement or arousal (31.6%), but these problems were less frequently reported, resulting in overall prevalence estimates for distressing problems at 2.8%, 3.2%, and 2.5%, respectively. Reaching a climax too quickly was least commonly reported (3.9%) and was experienced as distressing by only 10.8% of women reporting it, resulting in overall prevalence for distressing early climax of <1%.

**Figure 1:**
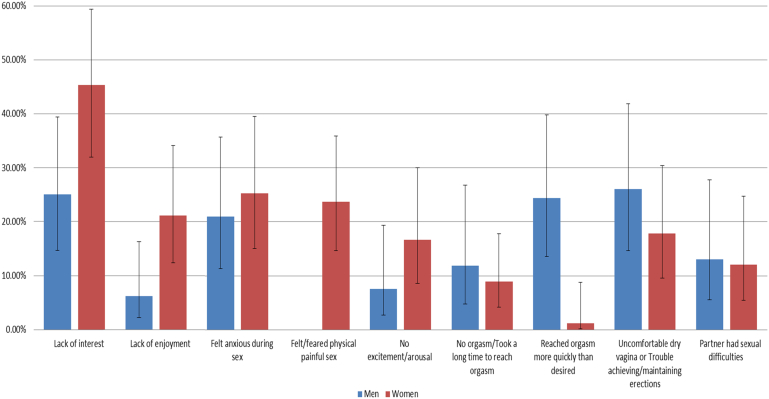
Reasons for avoiding sex among sexually active young people who reported avoiding sex because of a sexual difficulty.

Among young people who were sexually active in the last year, 6.3% of men and 6.8% of women said that they had avoided sex because of a sexual difficulty. Among young men (Figure 1), the most common reasons for avoidance were difficulty getting or keeping an erection, reaching a climax too quickly, and lack of interest (reported by 26.1%, 24.4%, and 25.1%, respectively, of all young men who said they had avoided sex). Among young women (Figure 1), the most common reasons for avoidance were lack of interest (reported by 45.5% of women who had avoided sex), followed by lack of enjoyment, anxiety, and pain (reported by 21.2%, 25.3%, and 23.7%, respectively, of women who had avoided sex).

### Help or advice seeking among sexually active participants

**Figure 2:**
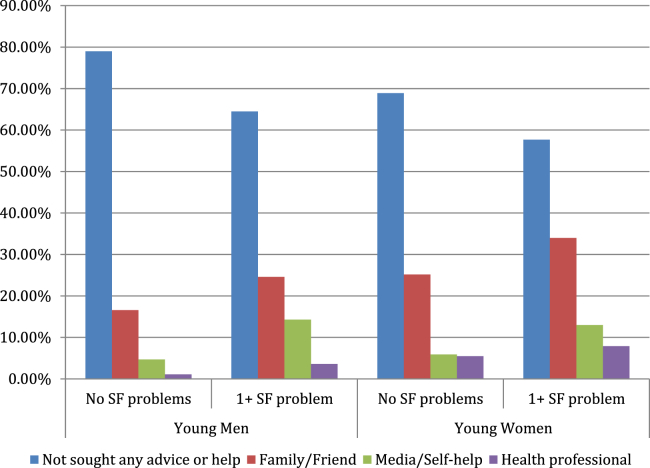
Proportion of young people who sought help or advice about their sex life by experience of sexual function problem and gender. SF = sexual function.

Overall, 26% (22.9–29.5) of sexually active men and 36.3% (33.1–39.7) of sexually active women had sought help about their sex life in the last year (last row, Tables 1 and 2). Figure 2 shows the proportions consulting the different sources, stratified by experience of sexual function problem. Those reporting one or more problem more commonly sought help compared with those reporting no problems (35.5% vs. 21% for men; *p* < .001 and 42.3% vs. 31.1%; *p* = .001). Where young people did seek help, family members and friends were the most common source followed by the media/self-help. Professional help was least commonly sought. Among young people reporting one or more sexual function problem, 3.6% (1.9–6.8) of men and 7.9% (5.8–10.6) of women had consulted professionals about their sex life in the last year.

### Distress and avoidance among young people who did not have sex in the last year

In total, 262 men and 255 women were sexually experienced (had ever had a sexual experience) but did not report having sex in the year before interview (Table 3). Just over one in six of these men (17.4%) and around one in eight of these women (12%) reported being distressed about their sex life, and around one in 10 (10%) of men and women said they had avoided sex because of sexual difficulties that either they or their partner experienced. There was no gender difference in reporting distress or avoidance.

## Discussion

These nationally representative data show that approximately one in 10 sexually active young men and one in eight sexually active young women report a distressing sexual problem lasting 3 months or more in the last year. The most commonly reported distressing problem among all sexually active men was reaching a climax too quickly (4.5%), and among young women, was difficulty reaching climax (6.3%). Over a third of men and more than four in 10 women reporting one or more sexual function problem had sought help, but rarely from professional sources. Among those who had not had sex in the year before interview, one in 10 young men and women said they had avoided sex because of sexual difficulties.

The strengths of this study are that it is based on a large population-based probability sample and addresses an important gap in the empirical evidence on sexual function problems among the young. Although the response rate of the overall survey (57.7%) represents a potential source of bias, the response rate among 16- to 44-year-olds was higher, at 64.8%. We have previously noted the recent general decline in survey response rates, coupled with more stringent methods for calculating them, and have also noted that our response rates are in line with other major social surveys in United Kingdom [25], [27]. Nonetheless, systematic bias in agreement to participate is possible, and we used survey weights to reduce this bias (see Methods). Items on sexual problems are sensitive, and self-reported data may be subject to recall bias and prone to under-reporting. We sought to minimize reporting bias by describing sexual function problems as “common difficulties” [22], by cognitively pretesting items [28], and by using computer-assisted self-interviewing [25].

Our data show sexual function problems are not uncommon in this age group. Estimates of the proportions of sexually active 16- to 21-year-old men and women reporting sexual function problems are not much lower than for the entire Natsal-3 population, 41.6% for men and 51.2% for women [22]. Several population-based studies have included and reported on younger age groups [10], [11], [12], [29] although comparison is limited by variation in survey methodology and categorization of both sexual problems and their severity. A recent Canadian study [13], for example, found that 50% of sexually active 16- to 21-year-old men and women reported a sexual problem, of whom, half reported associated distress, although the small, nonrandom sample and differences in definition suggest the need for caution in interpretation. Among young men, our prevalence estimate for erectile difficulties (7.8%) is midway between the 4.3% found in an Australian study of sexually active 16- to 19-year-olds [10] and 11% among sexually active 16- to 24-year-olds in a study in Portugal [12]. Our estimate of 13.2% for early ejaculation is slightly lower than the Australian study (15.3%) and much lower than the Portuguese study (40%). Among young women, our prevalence estimates for lack of interest (22%) and difficulty in reaching orgasm (21.3%) are slightly lower than those in the Australian study (36.7% and 29%, respectively) and comparable with rates of approximately 20% and 27% in a Swedish study of women aged 18–24 years [11].

It has been suggested that a proportion of problems in young people arise from a “practice effect” and that they disappear over time as young people gain confidence and experience. In support of this, O'Sullivan et al. [13] found that in young men, a longer period of sexual experience was associated with better erectile functioning and greater satisfaction with intercourse. On the other hand, a proportion of adults with sexual function problems report lifelong symptoms, in other words, symptoms that appeared at or before time of their sexual debut and have not subsided [8], [30]. A number of factors contributing to sexual difficulties are typically shaped in childhood and adolescence. These include inadequate sex education, difficulty in communicating about sex, anxiety about one's body or sexuality, and confusion or shame about one's sexual orientation or desires [31]. Sexual difficulties may also reflect the struggle to achieve positive sexuality within the confines of restrictive and gendered social norms, for instance, an acceptance that women should expect and endure pain [5]. The sexual double standard whereby women are censored and men rewarded for their sexual desire appears particularly resistant to cultural change [32], although recent research suggests variation in the extent to which young people assimilate these cultural scripts in their own relationships [33].

Over 25 years since the essay by Fine and McClelland [34] on the missing discourse of desire in sex education, young people continue to perceive a gap in their knowledge relating to psychosocial aspects of sex and often report feeling ill equipped to manage sexual intimacy. Natsal-3 data suggest that 42% of men and 47% of women wish they had known more about psychosexual topics at the time they first felt ready to have sex, including nearly 20% of men and 15% of women who wished they had known how to make sex more satisfying [35]. Similarly, in a mixed method study from New Zealand, students aged 16–19 years ranked “how to make sexual activity more enjoyable for both partners” and “emotions in relationships” among the top five topics they wished to know more about in school sex education [24]. While young people say they want to talk about pleasure, nonpenetrative alternatives to intercourse, and power relations in sexual relationships, school sex education tends to neglect these topics, the content instead reflecting the protectionist concerns of adults in authority [36].

Calls for inclusion of pleasure in sex education are not new [37]. The silence on sexual well-being from educative sources is filled by other sources such as friends and media; and, according to Natsal-3, nearly a quarter of young men cite pornography as one of their sources of information about sex [35]. Although some users perceive a positive impact on their sex life [38], pornography may lead to unrealistic and harmful expectations of sex among young men [39], potentially exacerbating sexual function problems. Sex education could do much to debunk myths, discuss pleasure, promote gender equitable relationships, and emphasize the key roles of communication and respect within relationships to militate against sexual problems.

The low proportion of young people with distressing problems who seek help or advice is perhaps unsurprising. Help seeking is uncommon, even among adults with sexual function problems [40]. Sex education could do much to address concerns, (1) by meeting gaps in knowledge; (2) by reassuring young people that problems are common and legitimate; and (3) by strengthening links to youth friendly services. Providers, in turn, need to be aware that young people attending for other sexual health needs (such as contraception and STI testing) may be struggling with concerns related to their sexual function. Given the prevalence of these concerns, it may be appropriate for providers to initiate discussion by asking about sexual function within a standard patient history, and future studies might evaluate the usefulness of this approach.

Without reliable data on young people's sexual function and well-being, calls for attention to this aspect of their sexual health can only be speculative. There is a pressing need for further youth-focused research exploring the scope of problems, their etiology and ramifications. In particular, there is a need for valid measurement tools that are specifically tailored to young people's issues.

In conclusion, if we wish to improve sexual well-being in the population, we need to reach individuals and couples as they embark on their sexual careers, to prevent lack of knowledge, anxiety, and shame turning into lifelong sexual difficulties. Our data provide a strong empirical impetus for taking this preventive action.

## Figures and Tables

**Table 1:** Experience of sexual function problems, and distress about these problems, among sexually active young men, aged 16–21 years

	% Reporting each sexual function problem		% Reporting each problem and distress about it		Of those reporting each sexual function problem, % fairly or very distressed about it	
Denominators^a^	854, 610		854, 610		281, 204	
	Percent	95% CI	Percent	95% CI	Percent	95% CI
Lacked interest in having sex	10.50	8.1–13.5	1.40	.8–2.5	13.20	7.2–22.8
Lacked enjoyment in sex	5.40	4.0–7.3	.90	.4–1.7	16.20	8.1–29.8
Felt anxious during sex	4.80	3.5–6.6	1.50	.8–2.7	30.40	17.9–46.6
Felt physical pain as a result of sex	1.90	1.1–3.4	.20	.1–.9	11.30	2.5–39.1
No excitement or arousal during sex	3.20	2.1–4.8	.80	.4–2.0	25.90	11.5–48.4
Difficulty in reaching climax	8.30	6.4–10.8	1.60	.8–3.0	19.20	10.5–32.4
Reached climax too quickly	13.20	11.0–15.7	4.50	3.2–6.3	34.20	25.5–44.1
Difficulty getting/keeping an erection	7.80	6.0–10.2	3.30	2.2–4.9	42.10	29.1–56.4
Experienced one or more of these	33.80	30.2–37.7	9.10	7.2–11.4	26.90	21.5–33.0
Sought help or advice for sex life	26.00	22.9–29.5				

CI = confidence interval.

a Denominator varies for each individual sexual function problem in this column. The unweighted and weighted denominator listed is for those that experienced one or more of these problems.

**Table 2:** Experience of sexual function problems, and distress about these problems, among sexually active young women, aged 16–21 years

	% Reporting each sexual function problem		% Reporting each problem and distress about it		Of those reporting each sexual function problem, % fairly or very distressed about it	
Denominators^a^	1,021, 553		1,021, 553		449, 242	
	Percent	95% CI	Percent	95% CI	Percent	95% CI
Lacked interest in having sex	22.00	19.3–25.0	5.30	4.0–7.0	24.00	18.4–30.6
Lacked enjoyment in sex	9.80	7.9–12.1	2.80	1.9–4.1	28.40	19.8–39.0
Felt anxious during sex	8.00	6.3–10.2	2.80	1.9–4.1	34.70	24.2–47.0
Felt physical pain as a result of sex	9.00	7.3–11.0	3.20	2.3–4.5	35.90	26.7–46.2
No excitement or arousal during sex	8.00	6.2–10.1	2.50	1.6–3.9	31.60	21.2–44.3
Difficulty in reaching climax	21.30	18.6–24.3	6.30	4.9–8.2	29.70	23.4–36.9
Reached climax too quickly	3.90	2.7–5.5	.40	.2–1.1	10.80	4.0–26.3
Uncomfortably dry vagina	8.50	6.7–10.6	2.20	1.5–3.4	26.20	17.5–37.2
Experienced one or more of these	44.40	41.1–47.8	13.40	11.3–15.9	30.20	25.7–35.1
Sought help or advice for sex life	36.30	33.1–39.7				

CI = confidence interval.

a Denominator varies for each individual sexual function problem in this column. The unweighted and weighted denominator listed is for those that experienced one or more of these problems.

**Table 3:** Proportion of sexually inactive 16- to 21-year-olds reporting distress about sex life, satisfaction with sex life, and avoidance of sex

	Men		Women	
Denominators	262, 165		255, 138	
	Percent	95% CI	Percent	95% CI
Distressed or worried about sex life	17.40	12.8–23.4	12.00	8.3–17.2
Avoided sex because of own or partner's sexual difficulties	10.10	5.5–17.9	10.70	5.4–20.1
Satisfied with sex life	34.60	28.5–41.3	32.20	26.2–38.7

CI = confidence interval.
